# Anti-High-Density Lipoprotein Antibodies and Antioxidant Dysfunction in Immune-Driven Diseases

**DOI:** 10.3389/fmed.2018.00114

**Published:** 2018-04-23

**Authors:** Javier Rodríguez-Carrio, Lourdes Mozo, Patricia López, Elena Nikiphorou, Ana Suárez

**Affiliations:** ^1^Area of Immunology, Department of Functional Biology, University of Oviedo, Oviedo, Spain; ^2^Instituto de Investigación Sanitaria del Principado de Asturias (ISPA), Oviedo, Spain; ^3^Department of Immunology, Hospital Universitario Central de Asturias, Oviedo, Spain; ^4^Academic Rheumatology Department, King’s College London, London, United Kingdom; ^5^Rheumatology Department, Whittington Hospital, London, United Kingdom

**Keywords:** anti-HDL, autoantibodies, high-density lipoprotein, antioxidant, autoimmunity, cardiovascular

## Abstract

**Introduction:**

Impaired high-density lipoprotein (HDL) levels and antioxidant functionality of HDL, mainly attributed to a decreased paraoxonase-1 (PON1) functionality, have been described in autoimmune conditions. In this setting, a role for humoral response in cardiovascular disease is emerging. This study evaluates the role of immunoglobulin G (IgG) antibodies against HDL and disease-related autoantibodies on HDL dysfunction in immune-driven diseases.

**Methods:**

Serum IgG anti-HDL antibodies, PON1 activity, and total antioxidant capacity (TAC) were quantified in 381 patients with different immune-driven diseases [18 mixed connective tissue disease (MCTD), 35 primary Sjögren syndrome (pSS), 38 systemic sclerosis (SSc), 33 ANCA-associated vasculitis (AAV), 60 diabetes mellitus 1, 29 autoimmune B12 deficiency/pernicious anemia, 29 primary biliary cirrhosis, 46 IBD/Crohn, 54 IBD/UC, and 39 celiac disease (CD)] and 138 healthy controls.

**Results:**

IgG anti-HDL antibodies were increased in MCTD, pSS, AAV, and inflammatory bowel disease (IBD) [Crohn and ulcerative colitis (UC)], even after correcting for total IgG levels, but not in organ-specific autoimmune diseases. Anti-HDL antibodies were negatively associated with PON1 activity in MCTD (*r* = −0.767, *p* < 0.001) and AAV (*r* = −0.478, *p* = 0.005), whereas both anti-HDL and anti-neutrophil cytoplasm antibod levels were related to an impaired PON1 activity and TAC in IBD/UC. In SSc, anti-centromere antibodies correlated PON1 activity. anti-*Saccharomyces cerevisiae* antibodies levels were negatively associated with PON1 activity (*r* = −0.257, *p* = 0.012) and PON1/TAC ratio (*r* = −0.261, *p* = 0.009) in IBD/Crohn. HDL dysfunction in CD was only related to anti-transglutaminase levels.

**Conclusion:**

IgG anti-HDL antibodies and HDL dysfunction are common hallmarks of systemic autoimmunity. Anti-HDL and disease-related autoantibodies account for the HDL antioxidant dysfunction in immune-driven conditions, mainly in systemic autoimmune disorders.

## Introduction

Systemic autoimmune disorders are commonly associated with an increased risk of cardiovascular disease (CVD) occurrence compared to the general population (1, 2). Different studies showed that traditional CV risk factors fail to fully account for this increased CV morbidity (3, 4), whereas chronic inflammation and immune dysregulation are thought to play a role (5, 6). However, the actual mediators remain unknown.

High-density lipoproteins (HDL) are pivotal in the prevention of atherosclerosis. HDL promote endothelial homeostasis by their role in the reverse cholesterol transport as well as by their antioxidant and anti-inflammatory activities (7). Altered levels of HDL and blood lipids have been described in systemic autoimmune diseases, and a paradoxical effect on CV risk (the so-called “lipid paradox,” whereby low lipid levels are associated with increased CV risk) is widely accepted (8, 9), although poorly understood. Similarly, impaired HDL functionality, mainly due to a decreased enzymatic activity of the calcium-dependent esterase paraoxonase 1 (PON1), has been reported in these conditions (10–12). Due to their association with CVD development, understanding changes in lipid levels and/or functionality as well as the underlying mechanisms, represents a major research topic.

In recent years, the hypothesis that autoantibodies can have a role in the pathogenesis of CVD has emerged from clinical studies (13–17). However, contradictory results (18, 19) suggest that although the humoral immune response can be linked to CVD development, the specific autoantibodies and their targets are yet to be elucidated.

Recent findings point to a detrimental interplay between autoantibodies and lipid profiles. Autoantibodies targeting HDL lipoproteins [immunoglobulin G (IgG) anti-HDL antibodies] and their components have been described in systemic lupus erythematosus (SLE) and rheumatoid arthritis (RA). Initially described in association with disease activity and antioxidant features (20, 21), our group has recently revealed an association among anti-HDL antibodies, lipid profiles, and lipoprotein functionality in SLE and RA (22–24). Interestingly, these antibodies were not related to traditional CV risk factors, hence confirming its role as non-traditional CV risk factors in this setting. Thus, these autoantibodies may be considered as orchestrators of the interactions between immunity and altered lipid profiles. However, with anti-HDL being documented in RA and SLE with similar clinical outcomes, these findings pose the question as whether these antibodies may be also detected in other immune-driven conditions.

We hypothesize that the HDL dysfunction associated with anti-HDL antibodies can be a common feature in immune-mediated diseases rather than a specific phenomenon of a single disease. Thus, the overarching aim of this study is to analyze the levels of anti-HDL antibodies in a broad range of different immune-mediated conditions. The specific objectives are (i) to analyze the levels of IgG anti-HDL antibodies in different immune-driven diseases, from systemic rheumatic to organ-specific autoimmune conditions, (ii) to study the associations between anti-HDL antibodies, lipid profiles, and HDL functionality, and (iii) to analyze the interactions between the IgG anti-HDL–HDL axis with clinical features and disease-related autoantibodies among diseases.

## Materials and Methods

### Patients and Controls

This study involved a cross-sectional sample of 381 individuals with different immune-mediated diseases recruited from the department of Clinical Immunology at the Hospital Universitario Central de Asturias (Asturias, Spain). Patients were recruited after a clinical appointment in the respective clinical departments (Internal Medicine, Rheumatology, Gastroenterology and Nephrology) at the same institution. Fasting blood samples, taken during the routine clinical examination at the clinical departments, were sent to the department of Clinical Immunology to be immediately processed and stored at −80°C until further analyses. A fresh aliquot was used for the determination of the panel of the disease-related autoantibodies for each condition (Table 1).

**Table 1:** Demographic features and blood lipid profiles of the individuals entered in this study.

	HC	MCTD	pSS	SSc	AAV	IBD/UC	IBD/Crohn	CD	PBC	DM1	B12/PA
	
*n*	138	18	35	38	33	54	46	39	29	60	29
**Demographic parameters**											

Female, *n* (%)	108 (78.2)	15 (83.3)	33 (94.2)	34 (89.4)	15 (45.4)	29 (53.7)	25 (54.3)	25 (64.1)	25 (86.2)	30 (50.0)	25 (86.2)

Age [mean (range)]	51.48 (25.33–80.00)	49.22 (21.00–84.00)	56.17 (28.00–79.00)	59.39 (29.00–87.00)	64.15 (27.00–85.00)	48.83 (19.00–83.00)	52.00 (18.00–85.00)	38.20 (18.00–77.00)	58.00 (40.00–84.00)	42.83 (18.00–71.00)	61.37 (29.00–85.00)

**Blood lipid profiles, mean ± SD**											

Total cholesterol (mg/dl)	201.01 ± 35.22	167.51 ± 31.29	184.35 ± 36.22	190.34 ± 36.92	202.02 ± 37.98	191.67 ± 40.87	174.84 ± 44.19	173.94 ± 36.08	195.12 ± 47.94	187.90 ± 34.84	187.89 ± 50.31

HDL-cholesterol (mg/dl)	59.67 ± 14.21	48.98 ± 10.95	62.20 ± 15.36	57.43 ± 16.85	61.40 ± 22.51	57.17 ± 17.60	49.05 ± 15.40	51.95 ± 16.60	57.33 ± 20.88	59.98 ± 17.61	56.81 ± 16.66

LDL-cholesterol (mg/dl)	123.56 ± 31.55	96.02 ± 32.22	113.16 ± 66.96	111.23 ± 32.51	115.68 ± 29.56	75.15 ± 36.07	66.78 ± 39.77	105.72 ± 34.67	107.14 ± 38.47	106.66 ± 31.57	111.42 ± 43.50

TC/HDL ratio	3.57 ± 1.02	3.52 ± 0.83	3.10 ± 0.91	3.51 ± 1.01	3.60 ± 1.18	3.61 ± 1.23	3.78 ± 1.14	3.70 ± 1.47	3.94 ± 2.17	3.39 ± 1.22	3.47 ± 1.09

Triglycerides (mg/dl)	84.47 ± 36.69	101.77 ± 44.88	89.8 5 ± 64.14	109.24 ± 51.95	133.91 ± 65.28	98.29 ± 50.04	117.52 ± 56.39	83.07 ± 46.18	113.67 ± 73.74	107.12 ± 96.93	122.58 ± 61.40

*Continuous variables are summarized as mean ± SD, unless otherwise stated. Categorical variables are summarized as *n* or *n* (%), as indicated*.

*AAV, ANCA-associated vasculitis; CD, celiac disease; DMI, diabetes mellitus 1; HDL, high density lipoprotein; MCTD, mixed connective tissue disease; PBC, primary biliary cirrhosis; pSS, primary Sjögren syndrome; SSc, systemic sclerosis; HC, healthy control*.

Disease diagnoses were confirmed by the corresponding consultant from each clinical department according to validated criteria: Alarcón-Segovia criteria for mixed connective tissue disease (MCTD) (25), 2002 EULAR classification criteria for primary Sjögren Syndrome (pSS) (26), 1980 ACR criteria for Systemic Sclerosis (SSc) (27), 1990 ACR criteria for the classification of vasculitis (28), criteria for primary biliary cirrhosis (29), American Diabetes Association criteria for type 1 diabetes mellitus (DM1) (30), classification criteria for gastritis and pernicious anemia (B12/PA) (31), Vienna/Montreal criteria for Inflammatory Bowel Diseases (IBD) (32, 33), and World Gastroenterology Organization guidelines for celiac disease (CD) (34).

A group of 138 matched healthy individuals (without any disease or treatment at the time of sampling) from the same population was simultaneously recruited as the healthy control (HC) group. Automated analysis of serum lipids was performed on fresh blood samples from all of the participants. Study approval was obtained from the Institutional Review Board (Comité de Ética Regional de Investigación Clínica) in compliance with the Declaration of Helsinki. All participants gave written informed consent prior to their inclusion in the study.

### Analysis of Autoantibodies

Antinuclear, anti-neutrophil cytoplasm, and anti-mitochondria antibodies [antinuclear antibodies (ANA), anti-neutrophil cytoplasm antibodies (ANCA), and anti-mitocondrial antibodies] were determined by indirect immunofluorescence on Hep-2 cells, ethanol/formalin fixed neutrophils, and rat liver/stomach/kidney (INOVA Diagnostics, San Diego, CA, USA), respectively. Anti-double strand DNA antibodies, myeloperoxidase, proteinase 3 (PR3), and transglutaminase IgA (TGA) antibodies were quantified in a chemiluminescent analyzer (ZENIT RA, Menarini Diagnostics, Firenze, Italy). ENA specificities (SSA/Ro, SSB/La, RNP, Sm, and Scl-70) were identified by line blot analysis (Euroimmun, Lübeck, Germany). An ELISA technique was used to quantify antibodies against gastric parietal cells, intrinsic factor (Menarini Diagnostics), glutamic decarboxylase (GAD), tyrosine phosphatase-related islet antigen 2 (IA2) (RSR Ltd., Cardiff, UK), and *Saccharomyces cerevisiae* [anti-*Saccharomyces cerevisiae* antibodies (ASCA)] (Wieslab, Malmö, Sweden).

### Determination of IgG anti-HDL Antibodies

Serum levels of IgG anti-HDL antibodies were measured by ELISA techniques as previously described (23, 35). Briefly, 96-well NUNC Maxisorp plates were overnight coated with 20 µg/ml human HDL (Sigma Aldrich, Germany) in 70% ethanol (test half) or ethanol alone (control half). After a blocking for 1 h (PBS 1% BSA), plates were washed and 1:50-diluted serum samples were incubated for 2 h at room temperature. Then, plates were washed with TBS (three times) and an alkaline phosphatase-conjugated anti-human IgG (1:1,000) (Immunostep, Spain) was added. One-hour later, the plates were washed twice with TBS and 1 mg/ml *p*-nitrophenylphosphate (Sigma) in dietanolamine buffer was added as substrate. Absorbance at 405 nm was recorded and signal from the control half of the plate was subtracted to that of the test half. Anti-HDL arbitrary units (AU) were calculated for each sample according to the standard curves (from pooled sera diluted 1:16 to 1:512).

Total IgG was quantified by conventional ELISA techniques and AU values obtained from the anti-HDL ELISA were corrected using total IgG levels (anti-HDL/IgG). To evaluate the burden of IgG anti-HDL positivity, the cutoff for an individual to be classified as “positive” was set using the 90th percentile found in the HC population (=90.78) (23, 35).

### Assessment of PON1 Activity

Serum PON1 activity was assessed according to the method described by Eckerson et al. with little modifications as previously described (23). Briefly, 300 µl of freshly prepared 1 mM paraoxon (Sigma) in 50 mM glycine buffer containing 1 mM CaCl_2_ (pH 10.5) was incubated with 7.5 µl of serum samples in 96-well NUNC Maxisorp plates for 20 min at 37°C. Production of *p*-nitrophenol was monitored at 405 nm. A unit of PON1 activity was expressed as micromoles of *p*-nitrophenol produced per minute and per milliliter of serum. Quality controls were included in each plate to correct for interassay variations.

### Analysis of Total Antioxidant Capacity (TAC)

Serum TAC was measured by means of a colorimetric assay based on the cupric reducing antioxidant capacity (CUPRAC method). A commercial kit (TAC Assay Kit, Sciencell Research Laboratories) was used following the instructions provided by the manufacturer. Serum TAC was expressed as millimolar Trolox equivalent units (mM T-Eq).

### Statistical Analysis

Continuous variables were summarized as median (interquartile range) or mean ± SD, whereas *n* (%) was used for categorical ones. Differences among groups were analyzed by Kruskal–Wallis (with Dunn–Bonferroni correction for multiple comparisons tests) or Mann–Whitney *U* tests, as appropriated. Spearman’s rank test was used for correlations, whereas χ^2^ tests were performed to analyze the distribution of categorical variables. Multiple regression analyses were carried out to evaluate the potential simultaneous effect of different independent factors on a dependent variable. Variables were log-transformed to achieve normal distribution prior to multiple regression analyses and *B* coefficients and 95% confidence intervals (CI) were computed. Significance was assumed at a *p*-value of <0.050. All statistical analyses were performed under SPSS 22.0 and GraphPad Prism 5.0 for Windows.

## Results

### Levels of IgG anti-HDL Antibodies in Immune-Mediated Diseases

**Figure 1:**
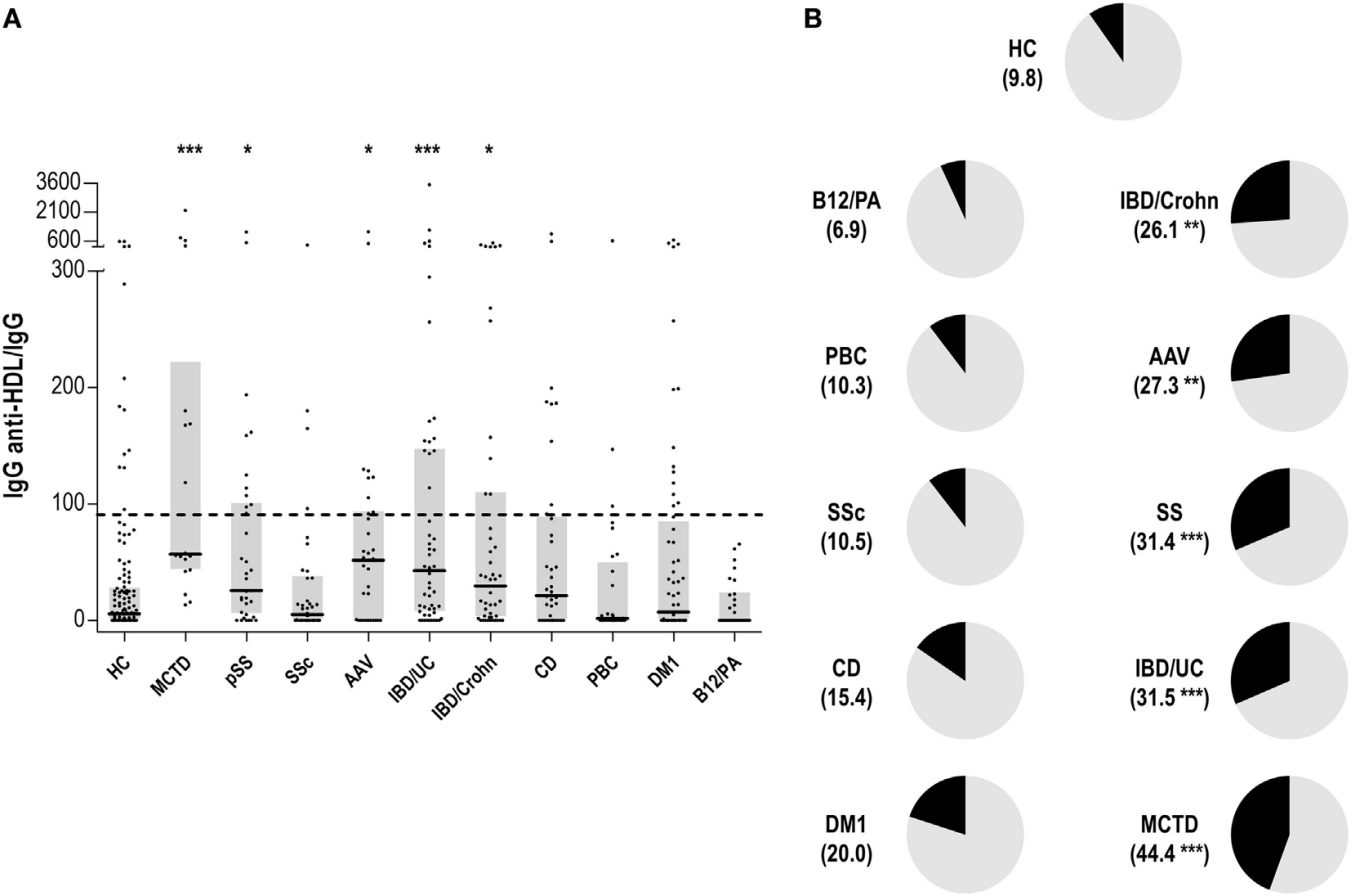
Immunoglobulin G (IgG) anti-high-density lipoprotein (HDL) antibodies in different autoimmune conditions. **(A)** Serum levels of anti-HDL/IgG antibodies in different autoimmune conditions and healthy control (HC). Each dot represents one subject, whereas horizontal bars represent the median value and gray boxes delimit 25th and 75th percentiles. Horizonal dashed lines represents the value of 90th percentile of anti-HDL/IgG in the HC group (=90.78). Differences against HC group were assessed by Kruskal–Wallis test and Dunnett correction for multiple comparisons test. **(B)** Positivity of anti-HDL antibodies in autoimmune conditions. The frequency of individuals classified as anti-HDL positive (using the 90th percentile found in the HC group as reference) is shown for each condition between parenthesis and represented as the black section of each graph. Differences compared to the HC were assessed by χ^2^ tests. Statistical significance is indicated as *p < 0.05, **p < 0.010, and ***p < 0.001.

The levels of IgG anti-HDL antibodies were quantified in serum samples from 138 HC and 381 patients with different immune-driven diseases (Table 1). Levels of IgG anti-HDL antibodies were found to be increased in MCTD [40.98 (164.65) AU, *p* < 0.001], pSS [19.72 (51.38) AU, *p* = 0.012], AAV [23.90 (42.26) AU, *p* = 0.030], CD [11.23 (31.63) AU, *p* = 0.050], IDB/Crohn [12.16 (53.03) AU, *p* = 0.020], and IBD/UC [17.34 (79.79) AU, *p* < 0.001] patients compared to those in HC [2.44 (12.92) AU]. Equivalent results were obtained when anti-HDL levels were corrected for total IgG levels (anti-HDL/IgG) (Figure 1A). According to the established cutoff, increased prevalence of anti-HDL positivity was found in MCTD, SS, AAV, IBD/Crohn, and IBD/UC (Figure 1B). IgG anti-HDL antibodies were not associated with age (*r* = −0.027, *p* = 0.765) nor with gender (*p* = 0.136) in HC or patients (both *p* > 0.050). Since a differential distribution of gender was observed in AAV, DM1, CD, IBD/C, and IBD/UC, a subgroup of HC with paired gender distribution was selected (Table 2). Differences in anti-HDL levels using this control population remained statistically significant (Figure S1 in Supplementary Material), therefore excluding the possibility that gender distribution could be a confounding factor.

**Table 2:** Subgroup paired analysis of the healthy control (HC) group.

	HC
*n*	76
Gender (f/m)	43/33
Age [mean (range)]	52.91 (25.33–73.00)
Total cholesterol (mg/dl)	203.17 ± 32.01
HDL-cholesterol (mg/dl)	55.05 ± 13.32
LDL-cholesterol (mg/dl)	125.46 ± 27.52
TC/HDL ratio	3.57 ± 1.02
Triglycerides	77.85 ± 32.63
Anti-HDL/IgG, 90th P	87.37

*No differences in gender were observed between this HC subgroup and ANCA-associated vasculitis (p = 0.285), DMI1 (p = 0.445), celiac disease (CD) (p = 0.437), inflammatory bowel disease (IBD) (p = 0.810), and IBD/UC patients (p = 0.745). This group was comparable in terms of lipid profile and anti-high-density lipoprotein (HDL) levels to the whole HC population (Table 1). Continuous variables are summarized as mean ± SD, unless otherwise stated. Categorical variables are summarized as n*.

Finally, the potential associations between anti-HDL/IgG levels and the blood lipid profile were studied. However, anti-HDL/IgG was not correlated with total cholesterol (*r* = −0.023, *p* = 0.801), nor with HDL (*r* = −0.002, *p* = 0.979) or LDL fractions (*r* = −0.035, *p* = 0.703) in HC, neither among immune-mediated diseases (all *p* < 0.050).

All these results revealed a specific increase in IgG anti-HDL antibodies in patients with systemic immune-mediated disorders, but not in organ-specific conditions.

### IgG Anti-HDL Antibodies and PON1 Activity

We wondered whether IgG anti-HDL antibodies could be associated to a decreased HDL functionality. To test this hypothesis, serum PON1 activity and TAC were measured.

**Figure 2:**
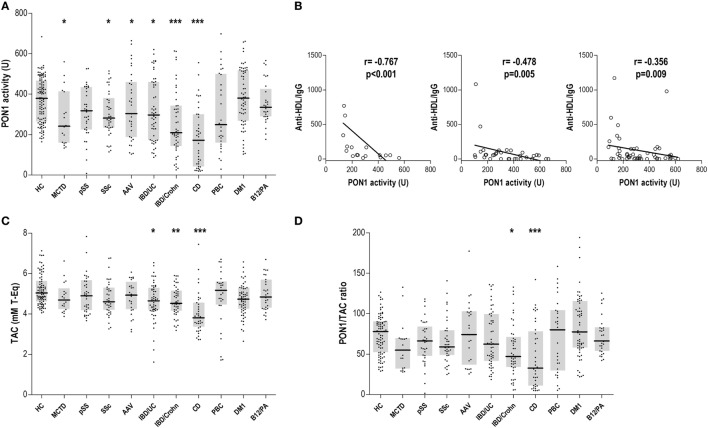
Paraoxonase 1 (PON1) activity, total antioxidant capacity (TAC), and PON1/TAC ratio in autoimmune conditions. **(A)** Serum PON1 activity, **(C)** serum TAC, and **(D)** PON1/TAC ratio were evaluated in autoimmune patients and healthy control (HC). Each dot represents one subject, whereas horizontal bar represents the median value and gray boxes delimit 25th and 75th percentiles. Differences against HC group were assessed by Kruskal–Wallis test and Dunnett correction for multiple comparisons test. Statistical significance is indicated as *p < 0.05, **p < 0.010, and ***p < 0.001. **(B)** Association between anti-high-density lipoprotein/IgG and serum PON1 activity in mixed connective tissue disease (MCTD), ANCA-associated vasculitis (AAV), and IBD/UC patients. Correlations were assessed by Spearman rank’s test.

Serum PON1 activity was decreased in MCTD, SSc, AAV, CD, IBD/Crohn, and IBD/UC patients (Figure 2A), suggesting an overlap between impaired PON1 activity and increased anti-HDL positivity. Interestingly, anti-HDL/IgG antibodies were negatively correlated with PON1 activity in MCTD, AAV, and IBD/UC (Figure 2B).

Total antioxidant capacity was decreased in patients with gut-related immune conditions (Figure 2C). Remarkably, anti-HDL levels were only associated with TAC in IBD/UC (*r* = −0.343, *p* = 0.011). However, a positive correlation between TAC and PON1 activity was observed in IBD/Crohn patients (*r* = 0.360, *p* = 0.014). The analysis of the PON1/TAC ratio (Figure 2D) revealed a different contribution of the PON1 activity to the total antioxidant status, being lower in CD.

Therefore, our findings demonstrate an antioxidant dysfunction in some immune-driven diseases. IgG anti-HDL antibodies may account for the impaired PON1 activity in some systemic conditions.

### IgG Anti-HDL Antibodies–PON1 Axis in Systemic Immune-Driven Diseases: Involvement of Disease-Related Autoantibodies

Since a notable heterogeneity was found among immune-driven diseases not only concerning the IgG anti-HDL levels observed but also regarding the association between the latter and serum PON1 activity, we aimed to evaluate whether disease-related autoantibodies (Table 3) can explain these discrepancies.

**Table 3:** Disease-related autoantibodies in the individuals entered in this study.

	Autoantibodies	Frequency
Mixed connective tissue disease (*n* = 18)	Antinuclear antibody (ANA)	18 (100.0)
	Anti-SSA	6 (33.3)
	Anti-ribonucleoprotein antibodies (Anti-RNP)	18 (100.0)
	Anti-double strand DNA antibodies (anti-dsDNA)	5 (27.7)
	Titer, mean ± SD	27.58 ± 57.49

Primary Sjögren syndrome (*n* = 35)	ANA	32 (91.4)
	Anti-SSA	27 (77.1)
	Anti-SSB	15 (42.8)
	Anti-RNP	2 (5.7)
	Anti-dsDNA	3 (8.5)
	Titer, mean ± SD	47.30 ± 3.60
	Anti-centromere	1 (2.8)

Systemic sclerosis (*n* = 38)	ANA	38 (100.0)
	Anti-SSA	2 (5.2)
	Anti-SSB	1 (2.6)
	Anti-RNP	0 (0.0)
	Anti-dsDNA	2 (5.2)
	Titer, mean ± SD	48.75 ± 14.21
	Anti-DNA topoisomerase I antibodies	7 (18.4)
	Anti-centromere	28 (73.6)
	1/320	1 (3.5)
	1/640	12 (42.8)
	1/1280	10 (35.7)
	1/2560	5 (17.8)

ANCA-associated vasculitis (*n* = 33)	ANA	8 (24.2)
	Anti-proteinase 3 antibod	13 (39.4)
	Titer, mean ± SD	116.28 ± 68.22
	Anti-myeloperoxidase	12 (36.4)
	Titer, mean ± SD	46.28 ± 49.59

IBD/UC (*n* = 54)	ANA	5 (9.2)
	p-ANCA	29 (55.7)
	1/80	8 (15.3)
	1/160	11 (21.1)
	1/320	6 (11.5)
	>1/650	4 (7.7)

IBD/Crohn (*n* = 46)	ANA	7 (15.2)
	p-ANCA	3 (6.5)
	anti-*Saccharomyces cerevisiae* antibodies	26 (60.8)
	Titer, mean ± SD	62.47 ± 49.78

Celiac disease (*n* = 39)	ANA	5 (12.8)
	Anti-transglutaminase antibodies	33 (84.6)
	Titer, mean ± SD	101.57 ± 79.48

Primary biliary cirrhosis (*n* = 29)	ANA	21 (72.4)
	Anti-centromere	7 (24.1)
	Anti-mitocondrial antibodies	26 (89.6)

Diabetes mellitus 1 (*n* = 30)	ANA	8 (13.3)
	Anti-glutamic acid decarboxylase antibod	34 (56.6)
	Titer, mean ± SD	671.74 ± 718.59
	Anti-islet antigen 2 antibodies	(31.6)
	Titer, mean ± SD	912.77 ± 1,465.64

B12/PA (*n* = 29)	ANA	3 (10.3)
	Anti-gastric parietal cell antibodies	23 (79.3)
	Titer, mean ± SD	54.09 ± 19.40
	Anti-intrinsic factor antibodies	7 (24.1)
	Titer, mean ± SD	134.25 ± 48.75

*The levels of different panels of disease-related autoantibodies were tested in the individual recruited for this study according to validated guidelines. The frequency of the autoantibodies was indicated as *n* (%) referred to the total of patients for each disease. The titers of some autoantibodies were expressed as mean ± SD or *n* (%) among patients positive for the corresponding antibody*.

**Figure 3:**
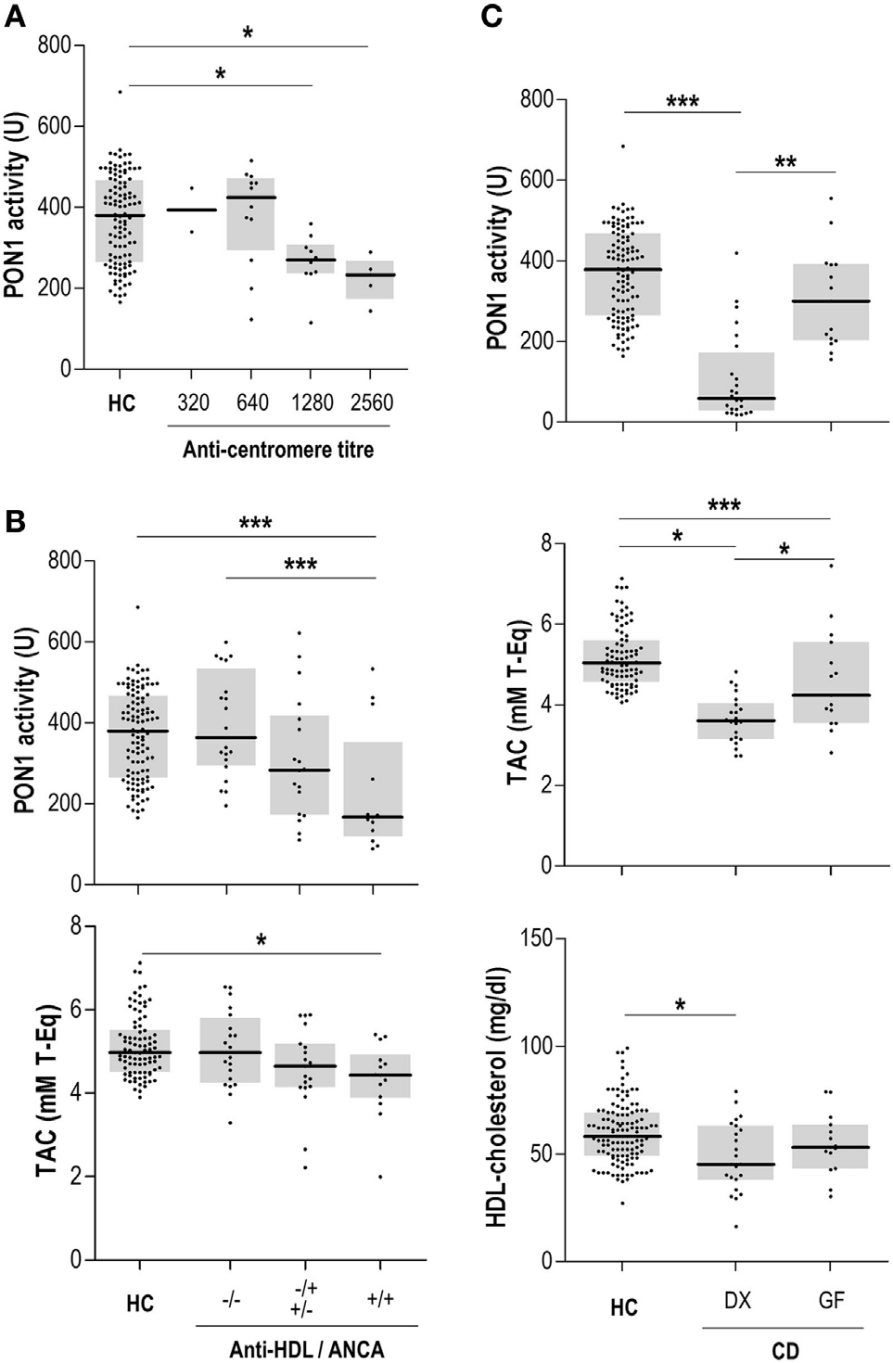
Anti-high-density lipoprotein (HDL) antibodies, disease-related autoantibodies, and paraoxonase 1 (PON1) activity. **(A)** Association between anti-centromere titer and serum PON1 activity in systemic sclerosis patients. **(B)** Association between anti-HDL and anti-neutrophil cytoplasm antibodies (ANCA) positivity and PON1 activity and serum total antioxidant capacity (TAC) in IBD/UC patients. **(C)** PON1 activity, serum TAC, and HDL levels in celiac disease (CD) patients recruited at diagnosis (DX) or those under a strict gluten-free diet (GF). Each dot represents one subject, whereas horizontal bars represent the median value and gray boxes delimit 25th and 75th percentiles. Differences were assessed by Kruskal–Wallis test and Dunn–Bonferroni correction for multiple comparisons test. Statistical significance is indicated as *p < 0.05, **p < 0.010, and ***p < 0.001.

Anti-HDL/IgG antibodies nor PON1 activity were associated with disease-related autoantibodies in MCTD, pSS, or AAV. Interestingly, a different picture was observed in SSc patients. No differences in IgG anti-HDL/IgG antibodies (*p* = 0.334) or PON1 activity (*p* = 0.253) were found between limited (*n* = 29) or diffuse SSc (*n* = 6) patients. However, ANA titer was negatively correlated with serum PON1 activity (*r* = −0.404, *p* = 0.012), this correlation being restricted to patients with a centromere pattern (Figure 3A). Additionally, SSc patients positive for anti-DNA Topoisomerase I antibodies exhibited diminished TAC compared to their negative counterparts [4.21 (0.60) vs 5.47 (1.39) mM T-Eq, *p* = 0.042].

Concerning organ-specific conditions, anti-HDL/IgG antibodies paralleled those of anti-glutamic acid decarboxylase antibodies (anti-GAD) (*r* = 0.246, *p* = 0.068) in DM1. Thus, anti-HDL positivity was slightly higher in anti-GAD positive DM1 patients (3/34, 26.4%) compared to their anti-GAD negative counterparts (3/22, 13.6%). Moreover, serum PON1 activity exhibited a non-significant negative trend with IA2 titer (*r* = −0.237, *p* = 0.079). No differences between DM1 recruited at onset (*n* = 6) and those recruited in later stages were found.

Gut-related conditions exhibited disease-specific patterns regarding the associations between the IgG anti-HDL-PON1 axis and disease-related antibodies. In Crohn patients, ASCA levels were associated with PON1 activity (*r* = −0.257, *p* = 0.012) and PON1/TAC ratio (*r* = −0.261, *p* = 0.009). On the other hand, PON1 activity was negatively correlated to ANCA titer (*r* = −0.382, *p* = 0.006) in IBD/UC. A clear additive effect on PON1 impairment was observed when both ANCA and anti-HDL autoantibodies were present (Figure 3B). This negative effect on PON1 activity was confirmed by multiple regression analysis: ANCA (*B*[95% CI], *p*: −0.326 [−0.284, −0.023], *p* = 0.022) and anti-HDL (−0.273 [−0.280, −0.002], *p* < 0.050). A similar detrimental effect was also observed for TAC (Figure 3B). Finally, PON1 activity and TAC were strongly decreased in CD, an association among anti-transglutaminase antibodies (anti-TGA) antibodies, HDL levels and antioxidant features being disclosed. Patients recruited at disease diagnosis (DX, *n* = 24) exhibited notable differences in serum PON1, TAC, and HDL levels compared with those with a longer disease duration following a strict gluten-free diet (GF, *n* = 15) (Figure 3C). These differences paralleled those found with anti-TGA levels [DX: 125.00 (178.78) vs GF: 15.80 (90.52) IU, *p* = 0.019].

Overall, anti-HDL IgG were not associated with disease-related autoantibodies, thus reinforcing the relevance of anti-HDL as novel biomarkers. Interestingly, anti-HDL and disease-related autoantibodies exhibited different associations with PON1 activity, HDL and PON1 among the different conditions.

## Discussion

Although a growing body of evidence supports a role for humoral immunity in CVD, the underlying mediators remain unclear. The results presented in this study point to the autoantibodies against HDL as the missing link between humoral response, lipoprotein dysfunction and oxidative stress in immune-driven diseases. Anti-HDL antibodies can be proposed as an explanation for the elusive hypothesis of the “lipid paradox.” Although initially described as a consequence of the inflammatory burden, further studies revealed that inflammation itself cannot totally account for the alteration in the lipid profile (9), thus suggesting the involvement of additional mediators. More recently, the concept of “lipid paradox” has been expanded to “HDL dysfunction,” since not only the levels but also the HDL functionality has been found altered in inflammatory diseases (36, 37). Thus, anti-HDL antibodies emerge as potential orchestrators of this phenomenon. Because of the role of lipoprotein dysfunction in CVD, a role for anti-HDL antibodies as biomarkers may be expected.

### Anti-HDL Antibodies in Immune-Mediated Diseases: Systemic vs Organ-Specific Conditions

A remarkable finding from our study is the difference in anti-HDL levels among different immune-driven diseases. The highest anti-HDL levels were found in systemic autoimmune rheumatic conditions (MCTD, pSS, and AAV) as well as in IBD, whereas increased anti-HDL levels were not observed in organ-specific autoimmune diseases. These results are in line with previous studies from our group and others, revealing a similar prevalence in RA and SLE (23, 24, 35).

Among systemic diseases, MCTD exhibited the highest prevalence of anti-HDL positivity. Interestingly, although CVD occurrence is increased in MCTD (38), the responsible mechanisms are unknown. Increased levels of disease-related autoantibodies have been found in MCTD patients with CVD (39, 40), thereby suggesting a role for humoral immune response in the CVD development in MCTD. However, these studies failed to disclose an association between these autoantibodies and markers of CVD. Instead, decreased apolipoprotein (Apo) A1, but not Apo B, was associated to impaired vascular homeostasis (39), stressing the relevance of HDL particles. Our results show a clear association between anti-HDL antibodies and the impaired PON1 activity in MCTD, thus strengthening the role of these autoantibodies in this condition. Moreover, MCTD exhibited greater alterations in surrogate markers of CVD than other connective tissue disorders, such as pSS or SSc (41) and different mechanisms of vascular damage between MCTD and SSc have been reported (42), which is in accordance with the differences in the association between IgG anti-HDL-PON1 axis and disease-related autoantibodies herein reported.

Another interesting finding from our study is the emergence of anti-HDL antibodies in gut-related diseases. Increased levels of anti-HDL antibodies were found in IBD, comparable to those in systemic autoimmune diseases. An increased CVD risk has been documented in IBD (43), especially during disease flares (44), hence suggesting the involvement of immune-driven mechanisms. However, although impaired lipid levels (45) and PON1 activity (46) have been reported in IBD, the underlying mediators are yet to be defined. Our findings expand the current knowledge in this scenario demonstrating a connection between anti-HDL and PON1 functionality in IBD. The differences in anti-HDL levels between ulcerative colitis (UC) and Crohn disease mirror that of the prevalence of subclinical atherosclerosis burden (47) and may be attributed to the shift toward Th2-response and humoral immunity present in UC compared to Crohn (48). Interestingly, HDL dysfunction in Crohn was similar to that of CD, in accordance with shared immunopathological features between these disorders (49). CD patients exhibited no increased anti-HDL levels, despite showing a notable impairment in PON1 activity and an increased pro-oxidant status. However, the alterations in PON1, TAC, and HDL levels were counteracted by a strict gluten-free diet, in parallel with a reduction of anti-TGA titers. Taken together, these findings may underlie the beneficial effects of a gluten-free diet on CVR factors in CD (50), something worth keeping in mind in clinical practice.

### Anti-HDL Antibodies and Lipoprotein Dysfunction: A Translational Approach for the Clinical Setting

CVR stratification and management in immune-driven diseases is suboptimal (51). A major criticism in the management approach over time has been the lack of a more holistic approach to patient care and a more index disease-centric care, with less attention to comorbid conditions or CVR. Moreover, the lack of a strong interphase between primary and secondary care, clinical pressures and health service infrastructures that challenge the management of factors and conditions beyond the index disease, along with routine unavailability of advanced imaging techniques for CVR stratification, all negatively influence the management of these conditions. Furthermore, available tools for CVR assessment have been demonstrated to be insufficient in systemic diseases (1, 52), as they are solely based on traditional CVR factors, hence emphasizing the urgent need of additional biomarkers. Taking into account our findings, anti-HDL antibodies may potentially represent a useful biomarker for addressing this unmet clinical need. Anti-HDL antibodies may improve the CVR management by identifying patients where lipoprotein dysfunction is observed and thus, capturing this pathological feature. Importantly, anti-HDL levels can be measured through conventional, operator-independent, and automatized laboratory techniques, hence representing a cost-effective option.

Overall, our findings delineate four different scenarios related to four different associations between the role of autoantibodies with the lipid profiles and HDL dysfunction. One scenario is composed by those diseases where lipoprotein dysfunction is solely associated with anti-HDL antibodies, such as MCTD or AAV. Previous results from our group and others suggest similar conclusions in SLE and RA (20, 22–24), with anti-HDL but not disease-related autoantibodies being associated with HDL dysfunction, indicating that this scenario may be restricted to systemic autoimmune diseases. Another group can be considered for diseases where blood lipid outcomes are determined by both anti-HDL and disease-related autoantibodies, such as IBD/UC or SSc. Moreover, some conditions exhibit alterations in the blood lipids that are only associated with disease-related autoantibodies, like IBD/Crohn or DM1. Finally, an independent group can be considered for those diseases where other (non-autoimmune) mechanisms are linked to HDL dysfunction, such as CD.

**Figure 4:**
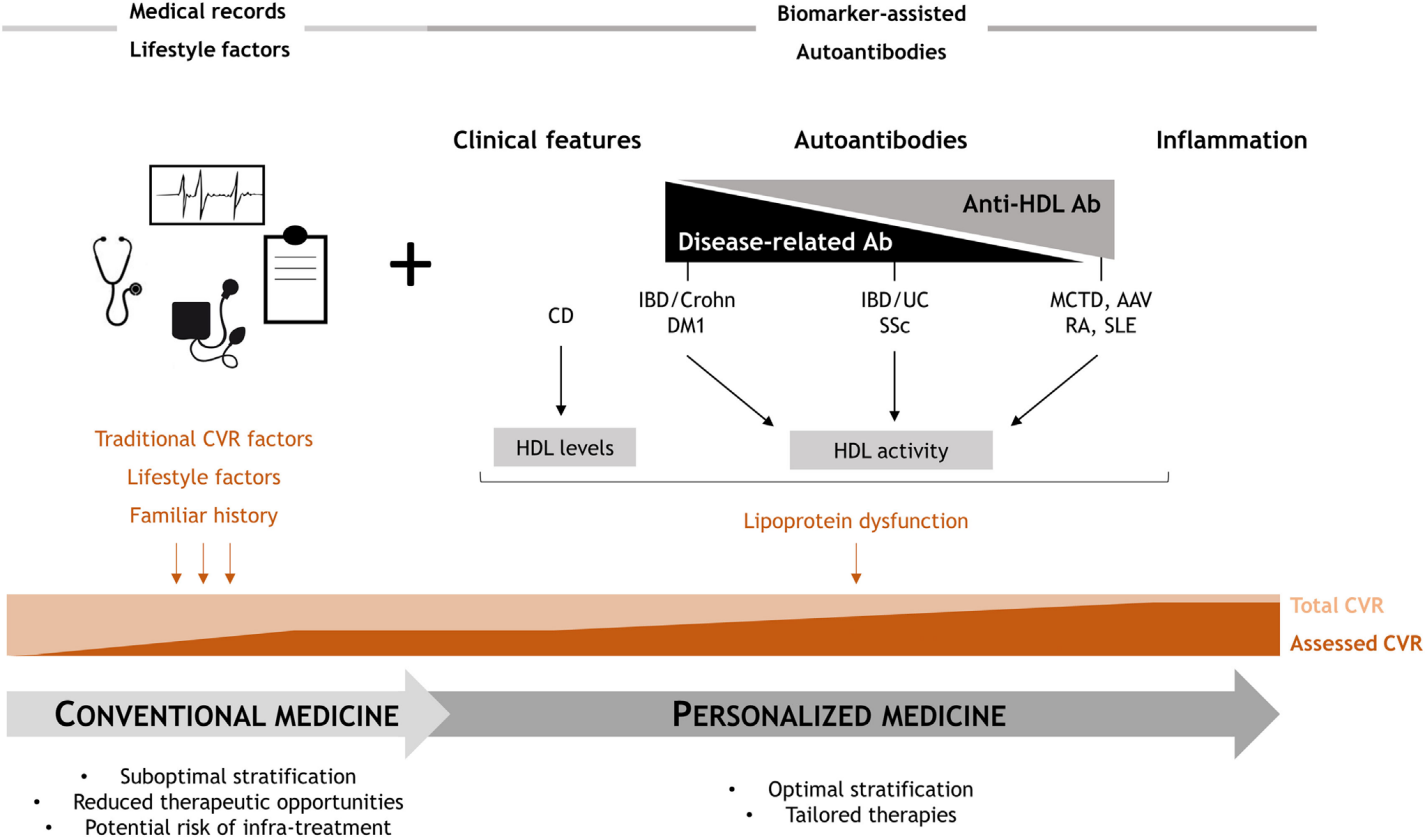
Overview of the study findings: clinical relevance. Conventional clinical CVR management provides a suboptimal risk stratification, since only a partial estimation of the actual risk is achieved. Anti-high-density lipoprotein (HDL) antibodies may cover this unmet clinical need, as they are able to account, at least in part, for the lipoprotein dysfunction. Depending on the role of anti-HDL and disease-related autoantibodies, four different scenarios can be proposed, hence providing a rationale for personalized medicine approaches to improve the current standards of care in these conditions.

The fact that different scenarios can be delineated paves the ground for the implementation of tailored strategies to complement the currently available CVR stratification strategies (Figure 4). Including a screening of anti-HDL antibodies in systemic diseases (first group) would be advisable to improve CVR stratification. Similarly, anti-HDL assessment together with disease-related autoantibodies should be performed in the second group of patients. On the contrary, a tight control of disease features, including disease-related autoantibodies screening, should be performed in the third group, since anti-HDL antibodies seem to not play a significant role. These different scenarios are in line with the concept of personalized medicine which is particularly relevant in those with complex clinical needs (Figure 4).

A number of limitations of the present report must be remarked. This study was designed as a proof of concept study to analyze the prevalence of anti-HDL antibodies in a wide range of immune-driven conditions beyond SLE and RA. Consequently, an in-depth analysis of the anti-HDL levels and specific clinical features or co-existing traditional CV risk factors in each condition was not conceived and surrogate markers of CVD were not evaluated in our analyses. Finally, the data did not allow for analysis of the treatments received in each group of patients. However, although this may be considered as a limitation, early studies by our group and others showed no major effects of immunomodulatory treatments (22–24, 53).

In conclusion, our findings revealed the presence of IgG anti-HDL antibodies in different immune-mediated diseases, with a strong connection to HDL dysfunction. Furthermore, higher prevalence of anti-HDL antibodies was observed in systemic rheumatic diseases. To the best of our knowledge, the emergence of anti-HDL antibodies is reported by our study for the first time in MCTD, AAV, and IBD. Our findings expand the current knowledge on the association between humoral immunity and CVD, demonstrating that anti-HDL antibodies represent a promising tool to account for the HDL dysfunction in immune-driven diseases. As such, their use as potential biomarkers in conjunction with traditional CV risk factor assessment may help better stratify CVR in a personalized medicine approach.

## Ethics Statement

Study approval was obtained from the Institutional Review Board (Comité de ética Regional de Investigación Clínica, Asturias, Spain) in compliance with the Declaration of Helsinki. Experimental and clinical procedures were performed in accordance with the recommendations of the Institutional Review Board. All participants gave written informed consent prior to their inclusion in the study.

## Author Contributions

JR-C performed most of the experimental procedures, carried out the statistical analyses, and drafted and edited the manuscript. LM was in charge of patients’ recruitment and clinical data collection. PL performed some experimental procedures and contributed to the interpretation of the data. EN contributed in the analyses, interpretation, and discussion of the results. AS conceived the study, designed the protocols, and drafted and edited the manuscript.

## Conflict of Interest Statement

The authors declare that the funders have no role in the study design, data collection, analysis, and interpretation of data nor in the writing of the report or in the decision to publish.

## Abbreviations

AAV, ANCA-associated vasculitis; AMA, anti-mitocondrial antibodies; ANA, antinuclear antibodies; ANCA, anti-neutrophil cytoplasm antibodies; anti-dsDNA, anti-double strand DNA antibodies; anti-GAD, anti-glutamic acid decarboxylase antibodies; anti-GPC, anti-gastric parietal cell antibodies; anti-IA2, anti-islet antigen 2 antibodies; anti-IF, anti-intrinsic factor antibodies; anti-MPO, anti-myeloperoxidase; anti-PR3, anti-proteinase 3 antibodies; anti-RNP, anti-ribonucleoprotein antibodies; anti-Scl70, anti-DNA Topoisomerase I antibodies; anti-TGA, anti-transglutaminase antibodies; ASCA, anti-*Saccharomyces cerevisiae* antibodies; AU, arbitrary units; CD, celiac disease; B12, autoimmune B12 deficiency; CV, cardiovascular; DM1, diabetes mellitus 1; HDL, high-density lipoprotein; IBD, inflammatory bowel disease; IgG, immunoglobulin G; MCTD, mixed connective tissue disease; PA, pernicious anemia; PBC, primary biliary cirrhosis; PON1, paraoxonase 1; pSS, primary Sjögren syndrome; SSc, systemic sclerosis; TAC, total antioxidant capacity; UC, ulcerative colitis.
