# Supplementary material for: Anti-High-Density Lipoprotein Antibodies and Antioxidant Dysfunction in Immune-Driven Diseases
Source: Front Med (Lausanne). 2018 Apr 23;5:114. doi: 10.3389/fmed.2018.00114 (PMC5925573; doi:10.3389/fmed.2018.00114)

**SUPPLEMENTARY MATERIAL**

**Anti-High Density Lipoprotein antibodies and antioxidant dysfunction in immune-driven diseases**

**Javier Rodríguez-Carrio^1,2^, Lourdes Mozo^2,3^, Patricia López^1,2^, Elena Nikiphorou^4,5^ and Ana Suárez^1,2^**

^1^ Area of Immunology, Department of Functional Biology, University of Oviedo, Asturias, Spain

^2^ Instituto de Investigación Sanitaria del Principado de Asturias (ISPA), Asturias, Spain

^3^ Department of Immunology, Hospital Universitario Central de Asturias, Asturias, Spain

^4^ Academic Rheumatology Department, King’s College London, London, UK

^5^ Rheumatology Department, Whittington Hospital, London, UK

**SUPPLEMENTARY FIGURES**

**Supplementary Figure 1: IgG anti-HDL antibodies in different autoimmune conditions and matched controls.**  Serum levels of anti-HDL/IgG antibodies in AAV, IBD/UC, IBD/Crohn, CD and DM1 patients and matched HC group (Table 2). Each dot represents one subject, whereas horizontal bar represents the median value and gray boxes delimit 25^th^ and 75^th^ percentiles. Horizonal dashed lines represents the value of 90th percentile of anti-HDL/IgG in the HC group. Differences against HC group were assessed by Kruskal Wallis test and Dunnet correction for multiple comparisons test. Statistical significance is indicated as * p<0.05, ** p<0.010 and *** p<0.001.


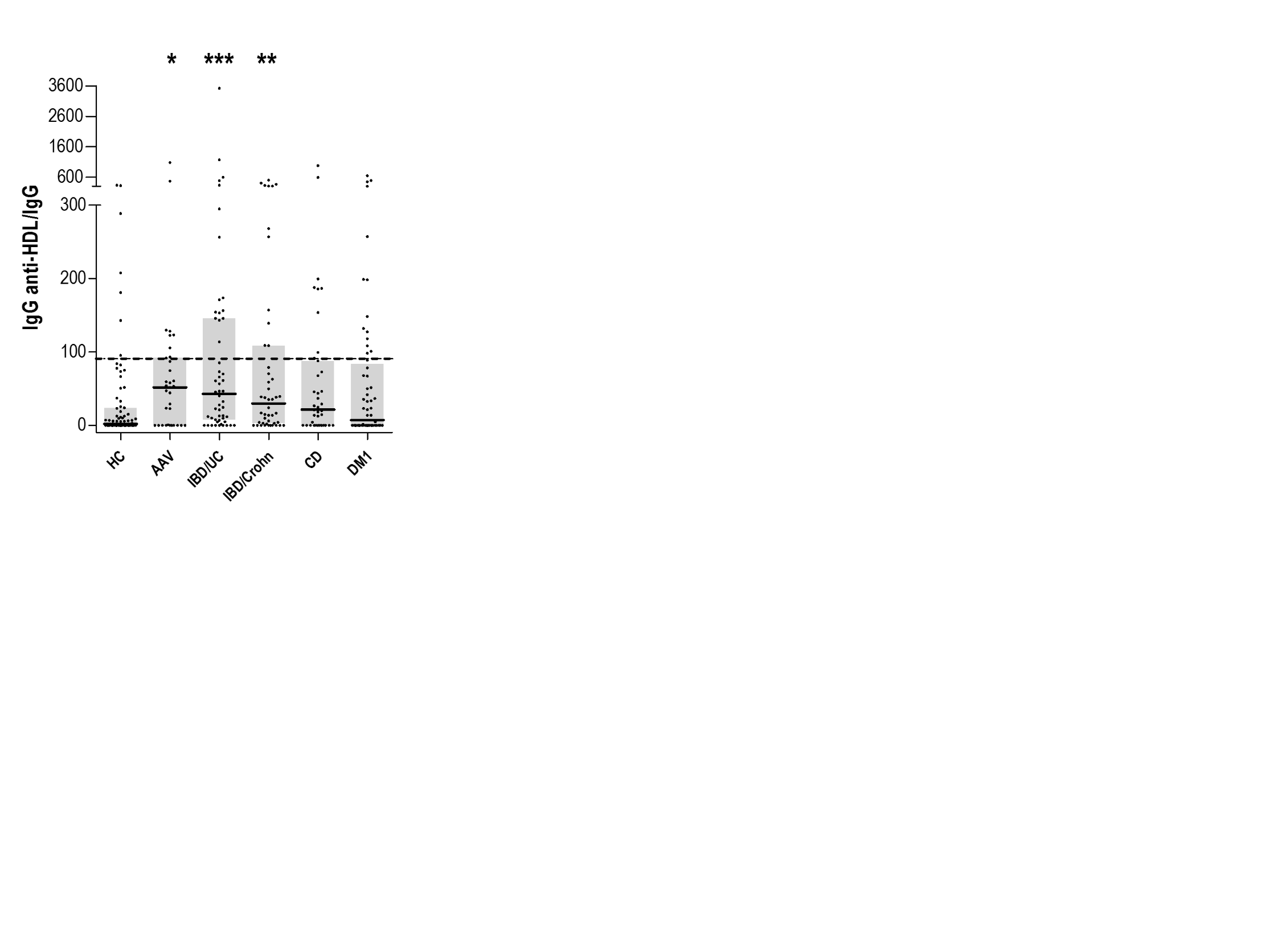

Supplement: Supplementary file 1 [file table_1.docx]
